# The Use of Camera Traps to Identify the Set of Scavengers Preying on the Carcass of a Golden Snub-Nosed Monkey (*Rhinopithecus roxellana*)

**DOI:** 10.1371/journal.pone.0087318

**Published:** 2014-02-03

**Authors:** Zhi-Pang Huang, Xiao-Guang Qi, Paul A. Garber, Tong Jin, Song-Tao Guo, Sheng Li, Bao-Guo Li

**Affiliations:** 1 College of Life Sciences, Northwest University, Xi'an, Shannxi, China; 2 The Nature Conservancy (TNC) China Program, Beijing, China; 3 School of Life Sciences, Peking University, Beijing, China; 4 Anthropology Department, University of Illinois, Urbana, Illinois, United States of America; 5 Institute of Eastern-Himalaya Biodiversity Research, Dali University, Dali, Yunnan, China; Oregon Health & Science University, United States of America

## Abstract

There exists very limited information on the set of scavengers that feed on the carcasses of wild primates. Here, we describe, based on information collected using a remote camera trap, carnivores consuming/scavenging the carcass of a wild golden snub-nosed monkey (*Rhinopithecus roxellana*) in the Laohegou Nature Reserve, Sichuan, China. During a 3 month behavioral and ecology study of a band of golden snub-nosed monkeys (March through May 2013), we encountered the carcass of an adult male (male golden snub-nosed monkeys weigh approximately 12–16 kg). After examining the dead monkey, we returned it to the death site and set out a camera trap to record the behavior and identity of scavengers. Over the course of 25 days, we collected 4145 photographs taken by the camera trap. Scavengers identified from these photographs include a masked civet (*Paguma larvata*), Asiatic black bear (*Ursus thibetanus*), large-billed crow (*Corvus macrorhynchos*) and the chestnut rat (*Rattus fulvescens*). No member of the golden snub-nosed monkey's social group, which was composed of approximately 120 individuals, was found to return to the general area of the death site. The masked civet fed principally on the face and intestines of the corpse at night, while the black bear consumed most of the body of the dead monkey during both the daytime and nighttime. These two taxa consumed virtually the entire carcass in one week. We suggest that the use of camera traps offers a powerful research tool to identify the scavenger community of a given ecosystem.

## Introduction

The ecology of scavenging offers important insight in advancing our understanding of the energy flow dynamics of natural ecosystems [1]–[3]. Scavenging is the consumption of animal remains that was killed by another individual or agent [1], [4]. A review of studies using experimental carcasses to measure scavenging efficiency indicated that across a range of climates and ecological systems, vertebrates consumed 75% of the available carcasses [1]. In addition, several recent studies using remote photography have demonstrated that a wide range of vertebrate species, including Raccoons (*Procyon lotor*), Virginia opossums (*Didelphis virginiana*) [2], Cougar (*Puma concolor*) [5], Giant Pandas (*Ailuropoda melanoleuca*) [6], jaguar (*Panthera oncaas*) [7], and western screech-owl (*Megascops kennicottii*) [8] that readily scavenge carcasses.

There exist several studies documenting scavenging behavior in wild primates, however, much of our present knowledge is limited to accounts of scavenging in early hominins [9]–[11], modern humans (Hadza) [12], and chimpanzees [13]–[18]. In contrast, very little is known regarding the set of scavengers that consume the remains of dead primates. In this study we describe, in detail, the coterie of scavengers that consumed the carcass of an adult male golden snub-nosed monkey (*Rhinopithecus roxellana*).

Golden snub-nosed monkey is an endangered species of Asian colobine whose distribution is restricted to temperate montane forests at 1000–4100 m above sea level across three isolated regions (i.e., Sichuan and Gansu, Shaanxi, and Hubei provinces) in central and southwestern China [19]–[22]. The largest remaining population includes approximately 10,000 individuals in Sichuan and Gansu provinces, with most golden snub-nosed monkey bands restricted to the Minshan Mountains [23]. Similar to its congeners (*Rhinopithecus. brelichi* and *R. bieti*), *R. roxellana* lives in large bands of 100 to 400 individuals [24]–[26]. Its social organization is described as a multilevel or modular society [27], and is composed of several independent harem or one-male breeding units (OMU). Each OMU consists of a single resident adult male, multiple adult and subadult females, juveniles, and infants [28]–[30]. Multiple OMUs and one to three all male units (AMUs, non-breeding units) travel, forage, feed, and rest together to form a band.

Two main hypotheses have been proposed to explain the evolution of a multilevel society or the spatial aggregation of several OMUs in *Rhinopithecus*. Zhang et al. (1999) suggested that the large size and coordinated travel of multiple OMUs that form a band may provide advantages in predator detection and predator defense [31]. Historically, the natural predators of snub-nosed monkeys are likely to include large cats (e.g., tiger: *Panthera tigris amoyensis*, leopard: *Panthera uncia*), canids (e.g., wolf: *Canis lupus laniger*, dholes: *Cuon alpinus*), the Asiatic black bear (*Ursus thibetanus*), and large raptors (e.g., Goshawk: *Accipiter gentilis*, Buzzard: *Buteo sp.*, Eagles: *Milvus korchun* and *Aquila chrysaetos*) [19], [31], [32]. Although some of these taxa have become regionally extinct over the past few decades [33], there exist two published reports of predation on snub-nosed monkeys, one involving a Goshawk (*Accipiter gentilis*) and the other a buzzard (*Buteo sp.*) [31], [32].

Alternatively, Grueter and van Schaik (2010) proposed that the threat from bachelor males who attempt to take over an OMU and oust the harem male may have selected for collective defense by multiple harem leaders against invading males [27]. Although infanticide is extremely rare in golden snub-nosed monkeys, there is one documented case of cannibalism by an adult male snub-nosed monkey following an infanticide event [34]. In addition, Xiang et al. (submitted) describe 59 instances in which harem males within a golden snub-nosed monkey band acted collectively to repel invasions by bachelor males [35]. In this study, all infants born into the band were sired by resident harem males [35]. Thus, collective defense may offer critical benefits to resident adult males in increasing their breeding tenure as well as reducing injury during aggressive encounters with invading males.

In the present study, we describe the use of a remote camera trap to identify the scavengers preying on the carcass of an adult male golden snub-nosed monkey. Our objectives were: 1) to determine whether the conspecifics would return to the death site and examine the carcass, 2) to identify animals that consumed the carcass, and 3) to determine how long it will take for the carcass to be completely consumed?

## Methods

### Ethics statements

The research protocols reported in this study adhered to the regulatory requirements of and were approved by the animal care committees of the Wildlife Protection Society of China (SL-2012-42). We obtained the agreements from the Provincial Forestry Department of Shaanxi and Sichuan, China, and their animal protection societies. Peking University obtained permission from the Sichuan Forest Department to conduct camera-trapping surveys and to monitor animal populations throughout numerous nature reserves in Sichuan Province, including Laohegou Nature Reserve (LNR) where this study was conducted. The camera-trapping survey and research protocols were approved by the administration of the LNR. Our research protocols adhered to the American Society of Primatologists principles for the ethical treatment of primates.

### Site and species

We studied the behavior, ecology, and social organization of golden snub-nosed monkeys inhabiting the LNR, Pingwu County, Sichuan, China from 20 Nov. 2012 to 15 July 2013. The LNR was established in 2012 by The Nature Conservancy (TNC) and the Sichuan Nature Conservation Foundation (SNCF). LNR represents the first land trust reserve established in China, is approximately 110 km^2^ in area, and protects critical habitat for the giant panda (*Ailuropoda melanoleuca*), Our 10 km^2^ study area within the LNR is composed of mountainous mixed broadleaf temperate forest (1400–3200 in altitude) and characterized by steep ravines and rugged terrain. We followed one semi-habituated band, the Gangou band, consisting of 12 OMUs and 1 AMU, approximately 120 individuals. Due to difficulties in following the monkeys across steep mountainous terrain, we conducted observations using a telescope at a distance of 200–600 m as part of a long-term and on-going study of the golden snub-nosed monkeys in LNR.

### Experimental procedure

On April 9^th^, 2013, we encountered a fresh carcass of a golden snub-nosed monkey while following the Gangou band. The distance from the carcass to the study group was 800 to 1000 m. We examined the carcass in the field to determine the sex, age, and cause of death, and then brought it back to the research station (Shabazi) for further examination. We believe the carcass was one to two days old when encountered, given that we had been in the same area three days earlier and had not observed the carcass. We preserved the monkey in a freezer at −20°C. On April 28^th^, we brought the carcass back to the original location and set an infrared-triggered camera trap (Reconyx™ PC900. Reconyx Inc., USA) to monitor the response of mammalian and avian visitors/scavengers to the carcass. The camera was set to work 24 h a day and to take 10 consecutive photograph at 1 s intervals once triggered. An invisible infrared flash was used to produce monochrome infrared photographs under low light conditions (i.e., dawn, dust and night).

### Data analysis

We examined all photographs taken by the camera, and used the time stamp on individual photographs to determine the date and time that conspecifics or other species entered the area. Consecutive photographs of an animal visiting the area were defined as a single visiting event, and photographs of the same species taken >10 min apart were considered as a separate visiting event. If the visiting animal was observed consuming the carcass, we recorded it as a scavenging event. We scored the date of the visiting or scavenging event as Di referring to the i^th^ day since the camera was operating (e.g., D1 referred to April 28^th^, the 1^st^ day the camera trap was operating).

## Results

Based on our examination of the golden snub-nosed monkey carcass, we concluded that this individual was an adult male, approximately 7 years of age, and weighing 12 kg. We found a 5 cm bite wound on his back, which we believe was the primary cause of his death. We also noted that his right maxillary canine and one mandibular incisor were missing.

**Figure 1:**
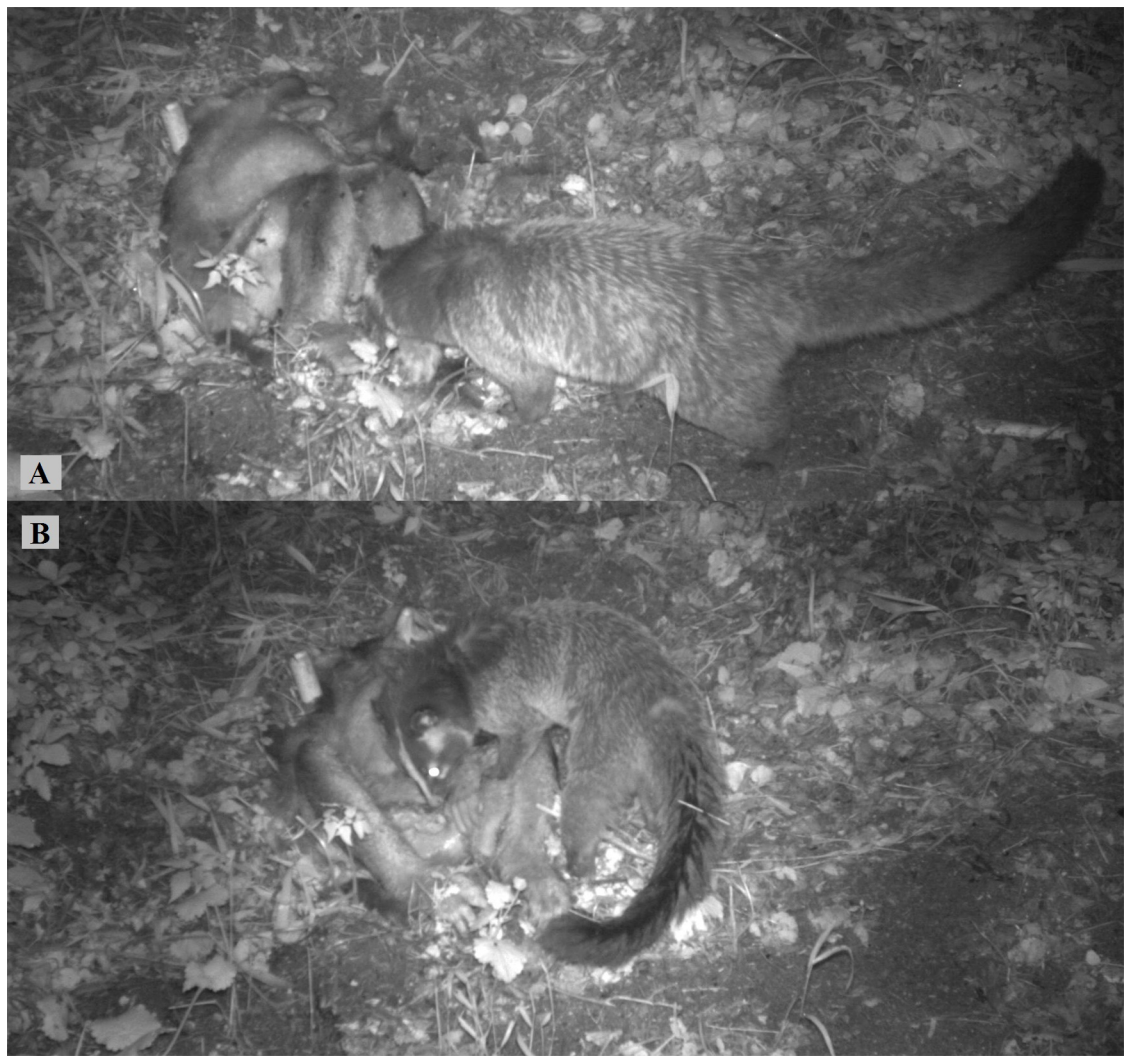
Masked civet consumed the intestines of a dead adult male golden snub-nosed monkey in Laohegou, (A) first time the carcass was detected and (B) consumption of the carcass (eating intestines).

The camera trap took 4,145 photographs during a 25-day study period. We identified four species that visited and consumed the carcass: the masked civet (*Paguma larvata*), Asiatic black bear (*Ursus thibetanus*), large-billed crow (*Corvus macrorhynchos*) and the chestnut rat (*Rattus fulvescens* Gray). A masked civet fist visited the carcass on D10 (21:00), sniffed it, and then left (Table 1, Fig. 1A). On D12, a masked civet visited on four occasions, three of these involved scavenging events. The civet spent approximately 1 min consuming part of the monkey's face (02:00), then consumed the intestines during a second visit (02:30, for 6 min), and on the third visit (04:30) spent an additional 46 min consuming the intestines (Fig. 1B). A civet returned again at 20:30, and spent 4 min consuming the remainder of the face and intestines. The civet left and never appeared again.

**Table 1:** Visiting and scavenging events on the carcass of a male golden snub-nosed monkey recorded using a camera trap in Laohegou Nature Reserve, Sichuan, China.

Date	Masked civet	Asiatic black bear	Chestnut rat	Large-billed crow	Total
D1			a0/2^b^		0/2
D2				0/1	0/1
D3			0/1		0/1
D4			2/4		2/4
D5			0/1		0/1
D6			2/2		2/2
D7			4/4		4/4
D8					
D9			0/2		0/2
D10	0/1				0/1
D11					
D12	4/4				4/4
D13				2/3	2/3
D14		4/4		2/2	6/6
D15		6/6		8/8	14/14
D16		4/4	1/1	5/5	10/10
D17		2/2		4/4	6/6
D18				3/3	3/3
D19				5/5	5/5
D20			0/1		0/1
D21					
D22					
D23				2/2	2/2
D24				3/3	3/3
D25				1/1	1/1
Total	4/5	16/16	9/18	35/37	64/76

a/b : no. scavenging event/no. visiting event.

**Figure 2:**
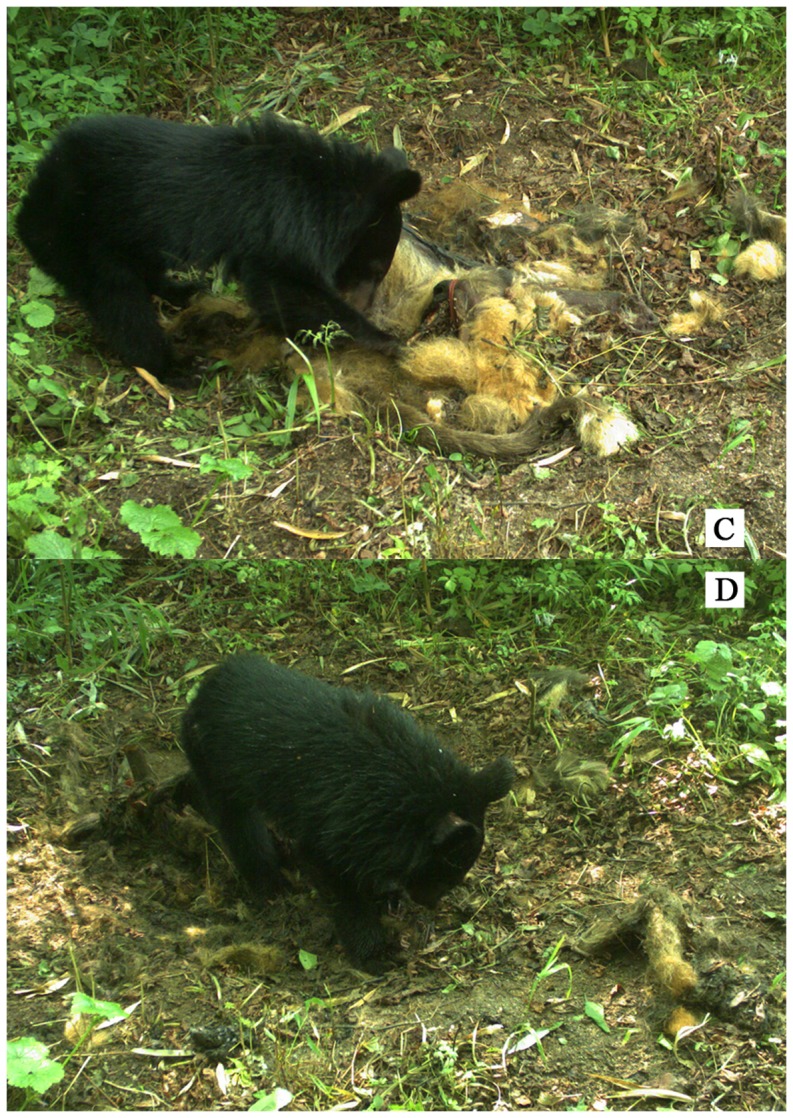
Black bear consuming the carcass of an adult male golden snub-nosed monkey in Laohegou, (C) first time the carcass was consumed and (D) complete consumption of the carcass.

A juvenile Asiatic black bear (*Ursus thibetanus*), approximately 1.5 years of age, visited the carcass during 16 events over a period of four consecutive days. Scavenging occurred during each visiting event and the average duration of scavenging was 10.7±6.0 min (range 2.5–23.4 min). The black bear first appeared on D14 (May 11, 2013) at 17:00, and consumed part of the carcass. Four visiting events were recorded on this day (Fig. 2C). The black bear came back during the next three days (six events on D15, four on D16, and two on D17). Visiting occurred during both the day and the night (including 02:00–03:00, 06:00–11:00, and 16:00–19:00, 22:00–23:00). Most of the carcass had been consumed by D17, and that was the last day the bear was observed to visit the area (Fig. 2D).

One large-billed crow (*Corvus macrorhynchos*) encountered and explored the carcass on D3, and then left. A crow came back each day from D13 through D25, with the exception of D20 to D22, for a total of 36 visiting events and 35 scavenging events. It fed on the carcass, and also consumed maggots that were present on the body. The crow visited the carcass only during daylight hours (06:00–17:00).

We recorded 18 visiting events and 9 scavenging events by a chestnut rat (*Rattus fulvescens*). The rat found the carcass on the evening of D1 but did not feed. A chestnut rat also was present on D3, D5 and D9, but no scavenging events occurred. A chestnut rat was present at the carcass on D4, D6 and D7, with 8 of 10 visiting events associated with scavenging. A chestnut rat also was found consuming something on the ground next to the carcass on D16 and D20. All visiting events occurred at night (20:00–03:00). We never observed a golden snub-nosed monkey in the vicinity or general area of the carcass.

## Discussion

Our manuscript presents the first detailed and systematic description of scavengers consuming the carcass of a golden snub-nosed monkey, despite the fact that these primates live in large groups of 100–300 individuals and researchers have studied wild bands for a period of 10–15 years. Reasons for such limited information include: 1) these primates have extremely large home ranges (15–20 km^2^) and live in dense forest and mountainous terrain which may limit the opportunities of researchers to encounter dead individuals. 2) Individuals that are wounded during intrasexual aggression tend to leave the band prior to death, and this is especially true in the case of adult males who compete to be the sole breeding male in a harem. The only other published reports of dead snub-nosed monkeys involve one infant *R. roxellana* from a provisioned population in the Qinling Mountains, Shannxi Province [41] and one infant black and white snub-nosed monkey (*R. bieti*) from Xiangguqing in Yunnan Province [42]. In addition, we observed a juvenile golden snub-nosed monkey with a serious wound on its back to leave a provisioned group and then disappear. It is likely that the carcass of this juvenile was rapidly consumed by scavengers over the course of several days and therefore was not found. Based on camera-trap data collected over 25 days, we identified four scavenger species (one rodent, two carnivores, and one corvid) consuming the carcass.

Although there are several reports of nonhuman primates reacting to the death of a conspecific and remaining in contact with the carcass for several days [36]–[41], we did not observe any conspecifics returning to the death site. This may reflect the fact that we had removed the carcass for a period of 20 days before returning it to the forest. In addition male golden snub-nosed monkeys leave their natal unit as juveniles, spend several years as a member of an all male unit, and only obtain reproductive opportunities by aggressively taking over an existing harem and ousting the harem leader or by forming a new harem by attracting adult females from other harems. Based on the wound present on this male golden snub-nosed monkey, we suspect his death was the result of fighting with one or more harem males. If he was an invading male, then band members might avoid the areas where threats from bachelor males have occurred.

The masked civet, Asiatic black bear, chestnut rat, and large-billed crow were the only vertebrate scavengers that consumed the corpse. Only one individual of each was observed to feed on the carcass during a scavenging event, with the crow visiting the carcass during the day, the masked civet and chestnut rat exclusively at night, and the Asiatic black bear visiting during both day and night. Moreover, the rat and crow were observed to consume only very small amounts of the carcass, and the masked civet consumed only the face and intestines. The black bear consumed most of the carcass. The corpse of the adult male snub-nosed monkey was consumed completely in one week.

We conclude, that scavenging represents an important component of all ecological communities and aids in the recycling of critical nutrients [1]. Our results broaden the understanding of the feeding ecology and behavior of scavengers inhabiting temperate mountainous forests in central China, and highlight the value of camera traps in systematically documenting difficult to observe events such as scavenging. We note that at the LNR, camera traps also have been used to document the scavenging activities of the giant panda (*Ailuropoda melanoleuca*). Wang et al. (2012) reported that the giant panda actively scavenged the carcass of a takin (*Budorcas taxicolor*) [6]. We plan to continue our research and expand our understanding of the community ecology of scavengers in this region of China.
